# The effects of task difficulty on gaze behaviour during landing with visual flight rules in low-time pilots

**DOI:** 10.16910/jemr.16.1.3

**Published:** 2023-03-20

**Authors:** Naila Ayala, Abdullah Zafar, Suzanne Kearns, Elizabeth Irving, Shi Cao, Ewa Niechwiej-Szwedo

**Affiliations:** University of Waterloo Ontario, Canada

**Keywords:** Eye movements, gaze entropy, area of interest, scanpath, visual scanning, aviation training, flight simulation

## Abstract

Eye movements have been used to examine the cognitive function of pilots and understand how information processing abilities impact performance. Traditional and advanced measures of gaze behaviour effectively reflect changes in cognitive load, situational awareness, and expert-novice differences. However, the extent to which gaze behaviour changes during the early stages of skill development has yet to be addressed. The current study investigated the impact of task difficulty on gaze behaviour in low-time pilots (N=18) while they completed simulated landing scenarios. An increase in task difficulty resulted in longer fixation of the runway, and a reduction in the stationary gaze entropy (gaze dispersion) and gaze transition entropy (sequence complexity). These findings suggest that pilots’ gaze became less complex and more focused on fewer areas of interest when task difficulty increased. Additionally, a novel approach to identify and track instances when pilots restrict their attention outside the cockpit (i.e., gaze tunneling) was explored and shown to be sensitive to changes in task difficulty. Altogether, the gaze-related metrics used in the present study provide valuable information for assessing pilots gaze behaviour and help further understand how gaze contributes to better performance in low-time pilots.

## Introduction

Modern aircraft cockpits present a complex human-machine interface where the success of the flight depends on the pilot’s ability to select relevant information from multiple competing stimuli. The dense visual field of instruments and displays conveys information about the status of the aircraft in real-time and must be closely monitored. Notably, 70-80% of global aviation accidents are caused by human error (58) with a major contributing factor proposed to be ineffective pilot monitoring of the plane, especially during dynamic phases of flight (i.e., take-off, final approach, and landing) (12; 51). It is crucial to understand a pilot’s information processing abilities underlying successful performance. Eye-tracking provides a non-invasive method that reveals discrete cognitive processes and strategies used to facilitate behaviour (3; 4; 16; 33; 47; 59; 68). For example, eye movement measures have been used as an important index for hazard perception in driving (16; 18; 74), human-machine interaction and usability assessment (34; 44; 49; 53), the development of visual strategies in athletes ( 66), and pilot behaviour assessment in aviation (55; 69). As such, the current investigation sought to examine the utility of gaze behaviour metrics to objectively characterize information processing in low-time pilots (fewer than 300 flight hours) during a landing task, and how it is altered by task difficulty.

The pattern of fixations and eye movements used to sample visual information in our environment is collectively referred to as gaze behaviour (35). Gaze behaviour is task dependent and tightly linked to the underlying perceptual, cognitive, and motor processes associated with the selection and processing of relevant sensory information (19). Therefore, it has a direct influence on the planning and execution of subsequent actions (30; 41). Studies have demonstrated that specific gaze metrics including total dwell time, fixation frequency, scan path length, average saccade amplitude and fixation duration are associated with performance in various laboratory paradigms (4; 33; 43; 47; 75). For instance, task relevant areas tend to be fixated longer as task difficulty increases suggesting that fixation location and dwell time are a proxy for the allocation of attention (4; 32; 42). Additionally, high performers show significant fixation biases toward task critical areas compared to low performers (32; 42). Fixation frequency, duration and scan path length tend to increase as a function of task difficulty, while saccade amplitudes decrease (2; 4; 32; 36; 52; 75) due to the increased scanning and processing required to problem solve and elaborate on longer solution sequences. Collectively, research to date supports that gaze measures provide objective insight into information processing that underlies differences in task difficulty and performance. However, a major limitation linked to these traditional gaze measures is that they are often time-averaging operations; thus, failing to make use of the information regarding the patterns and sequences of gaze behaviour. Such information may be critical to consider when evaluating gaze behaviour during complex occupational tasks and environments.

Gaze entropy represents one of the more established, advanced methods to quantifying the dynamic aspects of gaze behaviour (19). Entropy (measured in bits of information) is defined as the average information or uncertainty associated with choice (57). In the context of gaze behaviour, the complexity of an individual’s gaze pattern is governed by the number of regions (i.e., choices) that are fixated (8). These regions are characterized by defining the relevant areas of interest (i.e., AOIs) in an environment. There are two measures of gaze entropy that take into consideration the location and sequence of those fixations to compute gaze complexity, namely stationary gaze entropy (SGE) and gaze transition entropy (GTE). SGE is a measure of gaze dispersion that is computed over a given viewing period. The more equally distributed (i.e., wider gaze dispersion) fixations are across the environment (i.e., AOIs), the higher the entropy. Thus, a high SGE reflects an exploratory mode of visual attention whereas a low SGE reflects a focal mode of attention (59). GTE examines fixation sequence complexity through the analysis of gaze transition matrices (59). GTE assumes that fixation locations in a scan sequence are better predicted from current and previous locations through a conditional probability (71). High GTE indicates complex pattern of sequential scanning behaviour, which typically involves more frequent switching between more AOIs (4; 59). In contrast, low GTE reflects a more predictable scanning sequence with fewer fixation transitions between fewer AOIs. Note that a low GTE value can signal two different scenarios, either gaze has become more efficient and directed to relevant AOIs or it may indicate a failure to properly monitor the task environment. To interpret the entropy findings appropriately, it is important to ensure that SGE and GTE are examined in conjunction with traditional gaze metrics and behavioural performance. Such an approach provides a comprehensive account of gaze behaviour in complex environments, and potentially more insight into the underlying neurocognitive and sensorimotor processes (4).

Seminal research in the aeronautical domain has demonstrated an association between gaze behaviour and flying performance (for review see 28; 55; 73). For instance, deploying attention to the external environment in visual flight rules (VFR) conditions provides the operator with relevant visual information that facilitates successful landing (24; 29; 38; 56). Specifically, the optical splay angle is a visual cue that can be used to effectively align an aircraft with the runway centerline (9), whereas the runway length-width ratio can be used to regulate altitude (50). These findings are made more apparent during night landings, when these cues are less perceptible and consequently impact performance (38). In addition to the use of visual cues, other gaze specific parameters of attention allocation and information processing have been investigated through the use of traditional (i.e., dwell time, fixation frequency, fixation duration, saccade amplitude) and, more recently, advanced gaze metrics to characterize pilot gaze behaviour and the extent to which visual scanning changed with cognitive load (5; 6; 7; 21; 45; 62), situational awareness ( 20; 65; 64) and level of expertise (13; 28; 45; 55; 62; 73). The advantage of eye-tracking to probe pilot characteristics is that it provides real-time, objective data with minimal interruptions to the experiment or user, unlike questionnaires and probes.

The goal of the current study was to expand on previous work that specifically examined changes in gaze behaviour as a function of task difficulty (7; 23; 31; 62). A limitation in the previous literature is that cognitive load has been used interchangeably with workload, task load and task difficulty to describe the relationship between the demands imposed by a task and the availability of cognitive resources to perform that task. Here, we make a distinction between task difficulty and cognitive load. Task difficulty does not necessarily coincide with an increase in cognitive load, as the latter would be linked to task manipulations that increase the amount of information being held in working memory (54). Instead, task difficulty, which is germane to the current study, is tied to an increase in the sensorimotor control required to perform the task. For example, increasing the difficulty of a landing task by imposing high winds, shorter runways, steep approaches due to terrain require the pilot to impose higher sensorimotor control via corrective maneuvers during the operation of the plane in order to ensure a smooth, consistent, and safe flight (26). Notably, there is lack of consensus regarding the effects of task difficulty on gaze behaviour. While some studies showed task difficulty had no significant impact on pilot scanning behaviour or performance (23; 22; 39), other work showed increased fixation frequency (7; 31), more gaze transitions between task-relevant instruments (70), and longer dwell times on the runway (24; 56). The conflicting findings reported in previous work are likely a consequence of the various methods employed to characterize scanning behaviour as well as the wide range of flying experience seen across the recruited participants (i.e., commercial pilots, military pilots, individuals with no flight experience).

The current study aimed to clarify previous findings by examining the effect of task difficulty in low-time pilot performance. As such, we systematically manipulated task difficulty during a simulated landing scenario and examined traditional and advanced gaze metrics. The current investigation differs from previous work in two important respects. First, the pilot group recruited here involves low-time pilots who are at the early stages of their training (i.e., ab initio pilots). Previous work that focused on gaze and task difficulty examined experienced military and commercial pilots in advanced aircraft configurations (i.e., large, multi-engine aircrafts with glass cockpit displays) (13; 21; 23; 39; 64; 65; 69 ). Since gaze behaviour and sensorimotor control are significantly influenced by level of expertise (1; 15; 27; 55; 73), it is important to investigate the relationship between task difficulty and gaze behaviour in pilots, especially during their initial stages of training when they have little to no flight experience (i.e., ab-initio pilots). Such knowledge will advance our understanding on the relationship between eye movements and task difficulty, with specific implications for developing pilot training programs and evaluations. This is particularly relevant as improvements in the development of pilot competence in training are critical, since pilots are expected to progress more quickly from training through to airline and more advanced roles to address the expected international shortages of pilots (37). Second, the current investigation examined the utility of using a comprehensive set of eye movement analyses to characterize gaze behaviour dynamics as a non-invasive means to probe how gaze and, by proxy, information processing is impacted by task difficulty.

In line with previous findings (24; 31; 56; 70; 62), we hypothesized that an increase in task difficulty (i.e., turbulent weather conditions) would be associated with an increase in dwell time (specifically outside the cockpit), higher fixation rate, and a reduction in SGE and GTE. These findings are expected to underlie a greater need to devote more time and attention toward task relevant AOIs during turbulent conditions to extract critical information and ensure a safe landing.

## Materials and Methods

### Participants

Eighteen participants were recruited from the student and alumni populations at the University of Waterloo (14 males, 4 females; age range: 18-25 years, mean=20 years old, SD=2 years). All participants were either current aviation students or had graduated from the aviation program (number of flight hours range: 0-280, mean=64 hours, SD=91 hours; PC flight simulator experience range: 5-100 hours, mean=37 hours, SD=31 hours). All participants had normal or corrected-to-normal vision and had not been previously diagnosed with a neuropsychiatric/neurological disorder or learning disability. Participation in the study was voluntary, and participants received course credits as compensation. The study’s protocol was approved by the University of Waterloo Research Ethics Board Committee (#43238), performed in accordance with the 2008 Declaration of Helsinki, and consent was obtained prior to beginning the protocol.

### Apparatus

Participants sat in a height-adjustable chair with their chin placed in a chin rest. A 20-in LED monitor (85 Hz refresh rate 1920x1080 pixels, LG) was located at participants’ midline with a viewing distance of 50 cm and was used to present visual stimuli (i.e., the flying scenarios). A second computer monitor (85 Hz refresh rate, 1024x768 pixels, View Sonic) that was only visible to the experimenter was used to record eye position data using the EyeLink II eye-tracker (SR Research Ltd, Ottawa, ON, Canada) sampling at 500 Hz. Participants used a joystick and throttle (TCA Officer Pack Airbus Edition, ThrustMaster, USA), placed beneath the simulation display, to provide all necessary input commands. Prior to data collection, a nine-point calibration of the eye tracker was performed. An immediate follow-up validation of calibration accuracy was conducted to verify that the error was <1° for each point in the calibration grid. Stimuli presentation and behavioural data acquisition were controlled using Microsoft Flight Simulator 2020 (Asobo Studio, France).

### Scenario and task

**Figure 1.**
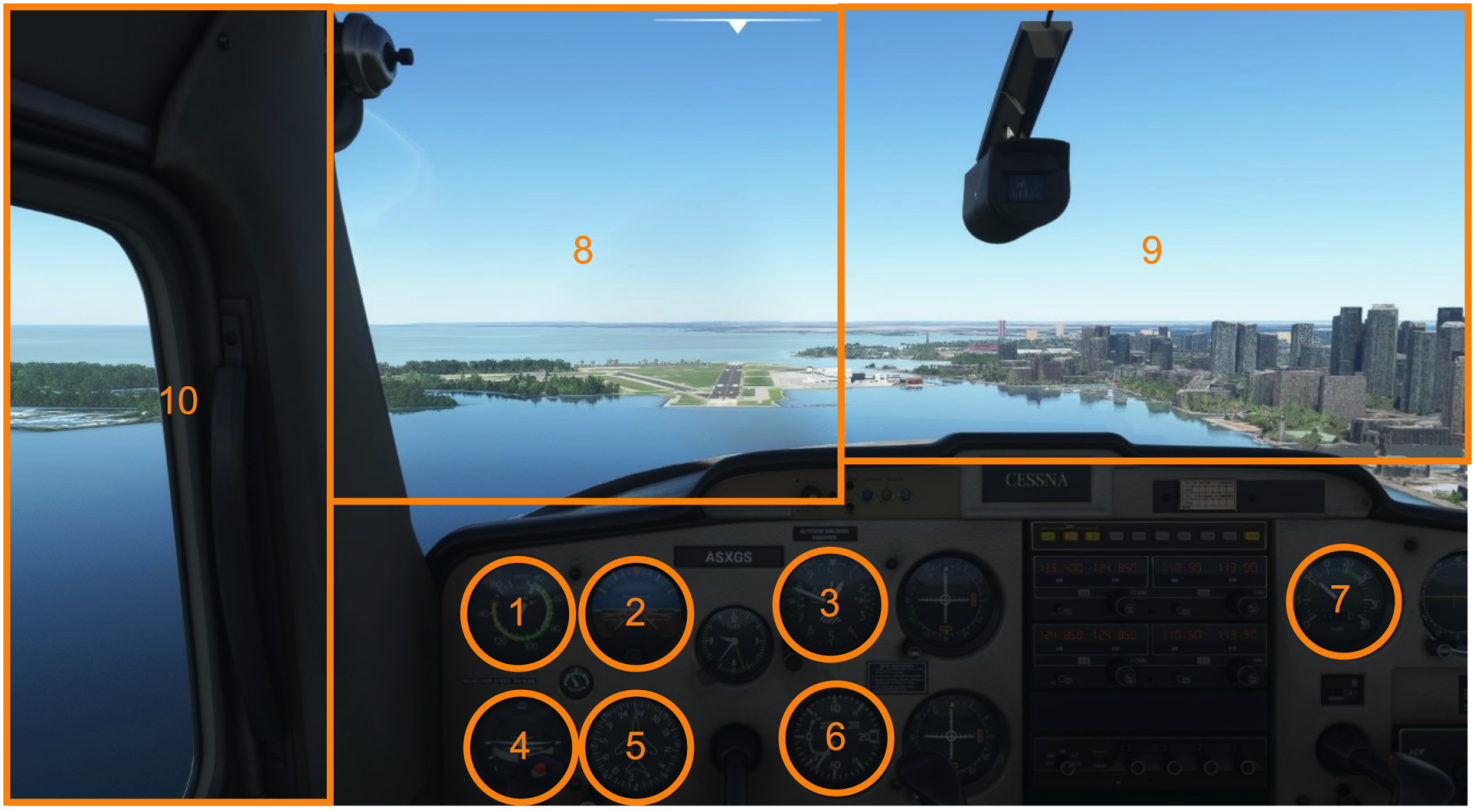
Illustration of the visual stimuli employed in the Microsoft Flight Simulator landing challenge environment. The participants point of view of the cockpit replicated that of a pilot flying a Cessna 152, pre-set for a straight-on approach to Billy Bishop airport, Toronto, Canada. The orange boxes represent the ten main areas of interest used in the gaze analyses. These include the airspeed (1), attitude (2), altimeter (3), turn coordinator (4), heading (5), vertical speed (6) and power (7) indicators, as well as the runway (8), horizon (9), and side window (10).

Participants were tested in a single session (approx. 90 minutes). A visual screening was first completed including a visual acuity test using the Bailey-Lovie chart and a stereoacuity test using the Randot Stereo test (Stereo Optical Company, Inc.). Prior to commencing the experimental trials, training was performed to familiarize the participants with the joystick and throttle controls (TCA Officer Pack Airbus Edition, ThrustMaster, USA). The experimental landing simulations were programmed in the Microsoft Flight Simulator landing challenge environment configured as a Cessna 152 (included steam-gauge instruments) flying into Billy Bishop airport (Toronto, ON, Canada). Participants were asked to complete a total of 20 customized landing challenges while their eye movements were recorded. The landing challenges were pseudo-randomized into 10 easy (i.e., high visibility and low wind conditions) and 10 difficult (i.e., high visibility and high wind conditions) trials. All participants received the exact same environmental configurations. Figure 1 shows a screen capture of the simulated scenario. Each trial was pre-set to start as a straight-on approach to the airport at an altitude of 1000 ft, 2.5 nautical miles away from the runway with flaps and trim set to zero, and at a starting speed of 120kts. The simulated landing task involved visual flight rules (VFR) where visibility is high and represents one of the most basic landing scenarios that ab initio pilots are faced with during training. This allowed for the extension of previous work that used similar paradigms and more advanced aircraft configurations (i.e., helicopter simulators, A320 flight simulators, larger aircrafts with glass cockpit displays) (13; 21; 64; 65). This was particularly important as the present work recruited low-time pilots.

At the start of each trial, participants were asked to look at a red dot marked on the monitor in order to standardize initial eye position. The trial was then manually initiated after a drift correction was completed by the examiner. The goal of the task was to land the plane as smoothly and accurately as possible relative to a blue landing ‘goal’ box near the start of the runway. The trial was terminated after the participant brought the plane to a complete stop, or if the landing was deemed unsuccessful (i.e., plane crash or plane landed off the runway).

### Data reduction

Eye movement data were analysed offline using the eye tracker’s Data Viewer software (ver 1.8: SR Research, Ontario, Canada). Eye-movement traces were visualized by the experimenter and played back at a slowed speed superimposed over the image displaying the gaming environment. The task environment was discretized within Data Viewer by organizing the gaming environment into ten areas of interest (AOIs) (Figure 1). The AOIs were manually defined to represent seven main gauges of interest within the cockpit including, airspeed (1), attitude (2), altimeter (3), roll coordinator (4), heading (5), vertical speed (6) and power (7). Three additional AOIs were also defined outside the cockpit including, the runway (8), the horizon (9), and the side window (10). Fixations found outside these AOIs were defined as a non-area of interest and excluded from the analysis (<3%). The AOIs were generated based on previous work and discussions with higher-hour pilots (i.e., >800 hours) and instructor pilots. In this way, the margin/border of each AOI is clearly explainable and allows for the entropy values to provide the most relevant and interpretable information from the scan patterns observed. This approach serves to be the most suitable for the current application since it is clear how the visual field should be grouped, and because there is strong ecological support from the piloting task and the cockpit design. In contrast, a grid/agnostic AOI approach would be most suitable for cases where researchers do not know how meaningful information in the visual field should be grouped. Eye movements were detected using a saccade detection algorithm implemented in Data Viewer with a 30°/s velocity threshold and an 8000°/s^2^ acceleration threshold. Fixations were defined as pauses between saccades that had a minimum duration of 80 ms (40; 67). The current study focused on primary saccades, thus microsaccades (<1°) were excluded from analysis (48). Trials with missing data (i.e., loss of signal >30%) (~5% of trials) and outliers for each of the dependent variables (i.e., >1.5 the interquartile range around the first and third quartiles) (~16% of trials) were removed.

### Entropy analysis

The entropy-based analysis was completed using the ten AOIs (Figure 1 ) that were discretized during pre-processing. Eye fixations in the ten AOIs were assigned a number from 1 to 10 indicating the AOI where the eyes fixated. A sequence of fixation locations was then generated for each trial. Custom scripts were written in Python to compute both SGE and GTE.

SGE was computed by first producing a vector, V, of length 10, where V_i_ was the total number of fixations at AOI i. V was then divided by the total number of fixations in the sequence, so that V_i_ was the probability of a fixation landing at AOI i. The probability vector V was then applied to Equation 1 (57).

Equation 1 $$H_{SGE}\left(V\right)=-\sum_{v\in V}^{}v\cdot \log\left(v\right)$$

GTE was computed by first creating a 10x10 transition matrix, M, where M_i,j_ was the total number of transitions from AOI i to AOI j. Each row, M_i,∗_, was divided by the sum of row i, so that M_i,∗_ represented the probability of fixation transition from AOI i to any of the ten AOIs. Finally, GTE was computed using Equation 2 (17), applying the transition matrix M and the probability vector V

Equation 2 $$H_{GTE}\left(M\right)=-\sum_{i=1}^{6}V_{i}\sum_{j=1}^{6}M_{i,j}\cdot \log\left(M_{i,j}\right)$$

### Performance measures

Performance dependent variables included success rate (%), completion time (sec), overall performance score (maximum of 2,000,000), landing accuracy (ft), ground roll (ft), and landing smoothness (fpm). These were all derived from Microsoft Flight Simulator 2020 (Asobo Studio, France). Success rate (%) was defined as the percentage of successful landing trials (i.e., participant landed on the runway without crashing) out of the total number of landing trials. Unsuccessful trials were automatically detected by Microsoft Flight Simulator when an aircraft either crashed or landed off the runway. Notably, these trials were not analyzed further for performance or gaze measures due to their rare occurrence (<2% trials). All other parameters reported are based on successful landing trials. Completion time (sec) was defined as the duration from landing challenge onset to landing challenge offset. Landing challenge offset was automatically determined based on when the plane came to a complete stop on the runway. Overall performance scores were generated by Microsoft Flight Simulator software for every landing using Equation 3, which was dependent on three sub-scores (i.e., landing smoothness, landing accuracy, and ground roll). Landing smoothness (fpm) was defined as the sink rate just before and at plane touchdown. Landing accuracy (ft) was defined as the distance between the centerline of the runway and the plane’s touchdown. Ground roll (ft) was defined as the distance between the center of the touchdown zone to the plane’s full stop.

Equation 3 OverallPerformance=(Accuracyscore+GroundRollscore)*LandingSmoothnessscore

## Results

Participants’ behavioural performance and eye movements were analyzed while they were performing the landing task. All performance measured were provided by the gaming software at the end of every trial. Raw eye-movement data were provided by EyeLink software for the duration of each trial. The main hypothesis was tested using a one-way repeated measures ANOVA with task difficulty (easy, difficult) as the independent variable. Our analysis is divided into three parts. In the first part, the landing performance measures were examined as a function of task difficulty. In the second part, traditional gaze metrics were assessed as a function of task difficulty. In the third part, entropy-based gaze analyses were carried out as a function of task difficulty. All ANOVAs were performed with an alpha level set at 0.05. The Bonferroni post hoc correction for multiple comparisons was applied for all post hoc analyses to determine significant differences between variables.

**Figure 2.**
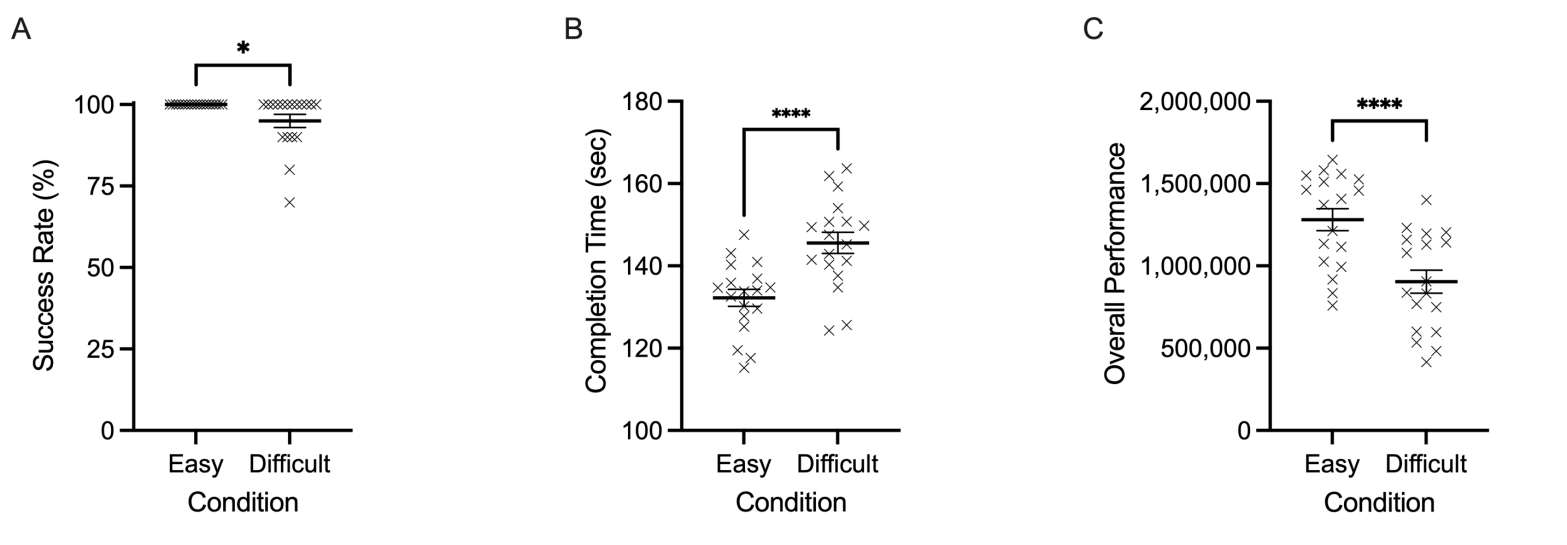
Individual data points and their respective group means for success rate (%) (A), completion time (sec) (B), and overall performance scores (C) are demonstrated for easy and difficult conditions. Error bars represent SEM. *p≤0.05, **p≤0.01, ***p≤0.001, ****p≤0.0001.

As expected, landing success rate was lower for difficult trials (mean= 95%, SD= 8%) compared to easy trials (mean=100%, SD=0%) (Figure 2A). Completion time (sec) produced a main effect of task difficulty, *F*(1,17)=105.740, *p*<0.0001, 𝜂_p_^2^=0.861. Figure 2B demonstrates how difficult trials (mean=146 sec, SD=11 sec) took significantly longer to complete than easy trials (mean=132 sec, SD=9 sec). Moreover, overall performance yielded a main effect of task difficulty, *F*(1,17)=113.456, *p*<0.0001, 𝜂_p_^2^=0.870. Overall performance scores were lower during difficult trials (mean=903898, SD=299342) compared to easy trials (mean=1280915, SD=284673) (Figure 2C).

**Figure 3.**
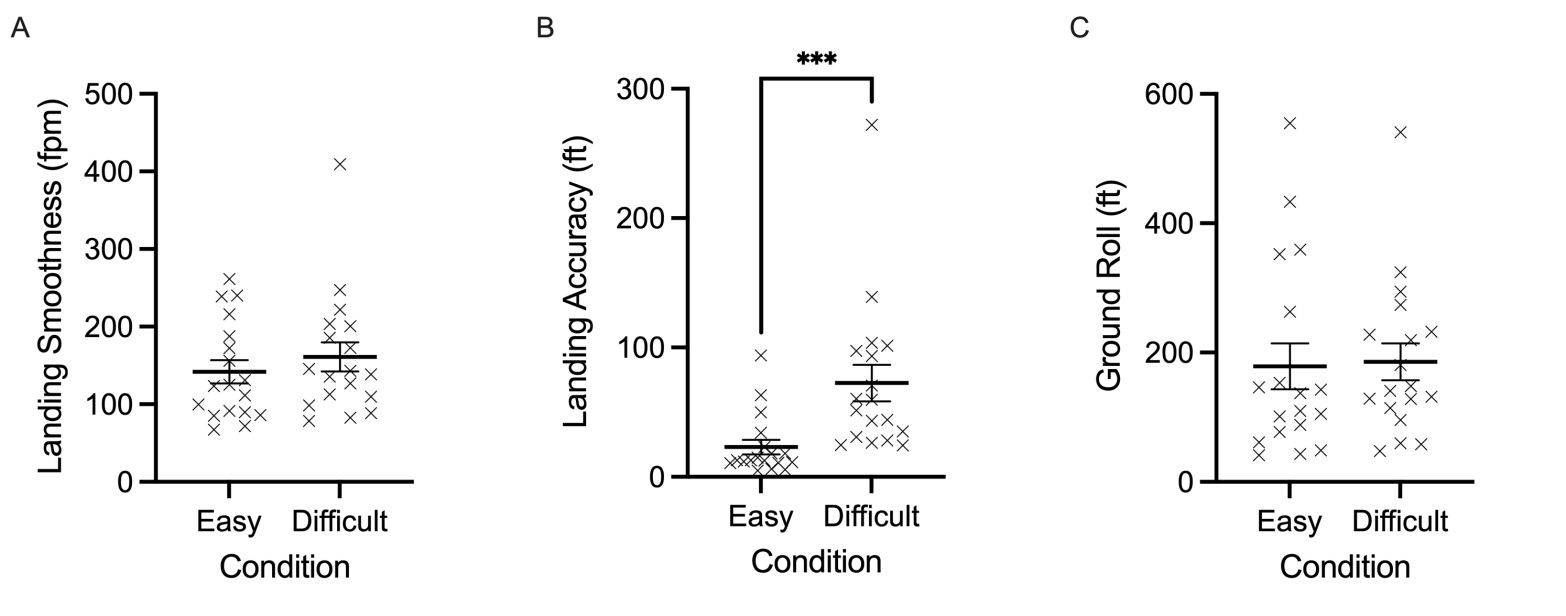
Individual data points and their respective group means for landing smoothness (fpm) (A), landing accuracy (ft) (B), and ground roll (ft) (C) are demonstrated for easy and difficult conditions. Error bars represent SEM. *p ≤0.05, **p ≤0.01, ***p ≤0.001, ****p ≤0.0001.

Landing smoothness (fpm) did not yield any significant effects F(1,17)=2.371, p=0.142 (Figure 3A). In contrast, landing accuracy (ft) yielded a main effect of task difficulty, F(1,17)=22.024, p<0.001, 𝜂p^2^=0.564. Results indicate that participants landed the plane with more lateral error (i.e., off-center) during difficult trials (mean=73 ft, SD=60 ft) compared to easy trials (mean=23 ft, SD=24 ft) (Figure 3B). Last, ground roll (ft) was not significantly modulated by task difficulty, F(1,17)=0.176, p=0.680 (Figure 3C).

### Traditional gaze measures

Traditional gaze-based analysis was completed using the ten AOIs (Figure 1) that were discretized during pre-processing using Data Viewer (SR Research, Ontario, Canada). Gaze dependent variables for the ten AOIs (Figure 1) included: dwell time (%), fixation rate (fixations/sec) and fixation duration (ms). Total scan path length (°), average saccade amplitude (°) and saccade amplitude (°) variability were calculated across all AOIs. Dwell time (%) was defined as the total duration spent within a given AOI, which was converted to a percentage (i.e., with respect to total time). Fixation rate (fixations/sec) was defined as the number of fixations that occurred relative to the total time spent completing the landing challenge. Fixation duration (ms) was defined as the average duration of all fixations within a given AOI. Scan path length (°) was defined as the sum of all saccade amplitudes. Last, average saccade amplitude (°) was the mean of all saccade amplitudes recorded, whereas saccade amplitude (°) variability was the within participant standard deviation of saccade amplitude (°). Means and standard deviations for all traditional gaze measures are reported in Table 1.

**Table 1.**
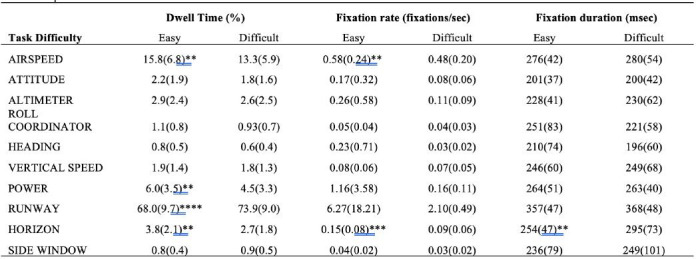
Traditional gaze values calculated for all areas of interest during easy and difficult conditions.

Note. Mean (standard deviation) values for all traditional gaze measures across all areas of interest (AOI) and task conditions (easy versus difficult). Significant changes between task difficulties and their corresponding dependent variable and AOI reported via *p ≤0.05, **p ≤0.01, ***p ≤0.001, ****p ≤0.0001.

**Figure 4.**
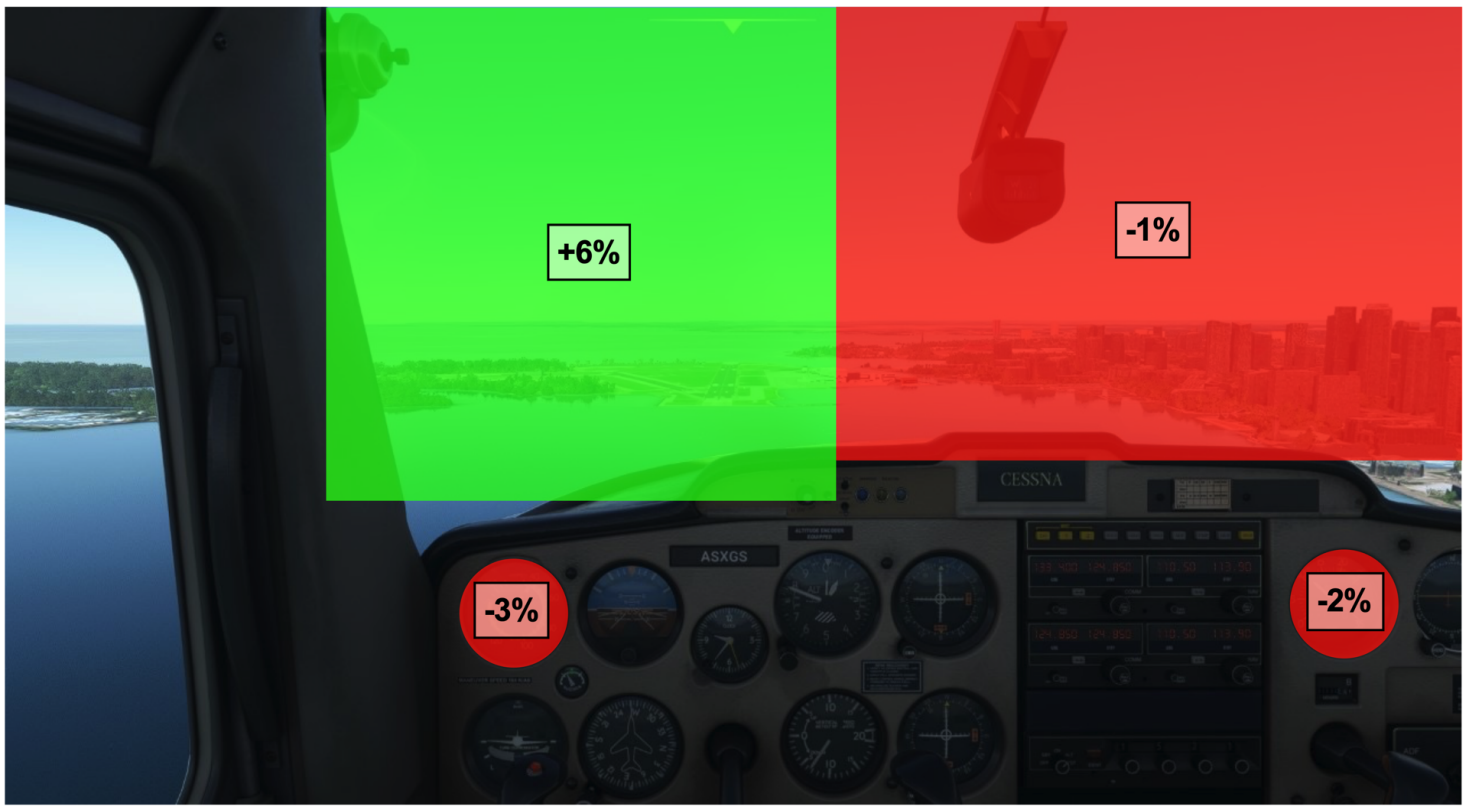
Illustration of the visual stimuli employed during landing and the corresponding change in dwell time (%) between easy and difficult conditions. Only significant changes in dwell time are displayed over their corresponding area of interest (AOI) (i.e., airspeed, power, runway, and horizon AOIs). Group mean differences (difficult-easy) are illustrated in red to indicate a decrease in AOI dwell time and green to indicate an increase in AOI dwell time.

Dwell time (%) revealed significant changes across several AOIs as a result of task condition. First, decreased dwell time was found for airspeed *F*(1,17)= 13.006, *p*=0.002, 𝜂_p_^2^=0.433; power *F*(1,17)=13.043, *p*=0.003, 𝜂_p_^2^=0.532; and horizon AOIs *F*(1,17)=13.702, *p*=0.002, 𝜂_p_^2^=0.446. Runway dwell time (%) was the only AOI to show a significant increase as a function of task difficulty, *F*(1,17)=37.559, *p*<0.0001, 𝜂_p_^2^=0.688. All other AOI’s did not reveal a significant change in dwell time, *Fs*(1, 17)<2.861, *ps*>0.117 (Table 1). Note that the significant changes in the distribution of attention (i.e., dwell time %) observed between easy and difficult conditions are illustrated in Figure 4.

Airspeed fixation rate demonstrated a main effect for task difficulty, *F*(1,17)= 13.688, *p*=0.002, 𝜂_p_^2^=0.446. Fixation rate within the airspeed AOI was higher during easy trials compared to difficult trials. Horizon fixation rate was also significantly modulated by task difficulty, *F*(1,17)=22.335, *p*<0.001, 𝜂_p_^2^=0.568. Easy trials had higher fixation rates within the horizon AOI compared to difficult trials. All other AOI’s were not significantly modulated by task difficulty, *Fs*(1,17)<1.436, *ps*>0.247 (Table 1).

Horizon was the only AOI where fixation duration was significantly modulated by task difficulty, *F*(1,17)=8.771, *p*=0.009, 𝜂_p_^2^=0.340. Fixation durations were longer during difficult trials compared to the easy trials. All other AOIs did not have significantly altered fixation durations, *Fs*(1,17)<2.177, *ps*>0.056. Lastly, scan path length was not significantly influenced by task difficulty, *F*(1,17)=0.001, *p*=0.974 (easy: 1205 °, SD=715°; difficult: 1200°, SD=546°). However, increased task difficulty was associated with a significant reduction in average saccade amplitude (easy: 4.96°,SD= 1.38°; difficult: 4.58°, SD=1.35°; *F* (1,17)=10.078, *p*=0.006, 𝜂_p_^2^=0.372) and saccade amplitude variability (easy: 6.53°,SD= 1.50°; difficult: 5.91°, SD=1.62°; *F* (1,17)=16.951, *p*=0.001, 𝜂_p_^2^=0.499).

### Entropy-based measures

SGE represents the dispersion of fixations and an overall uncertainty of fixating in a particular AOI at any given moment (Equation 1). A higher SGE value represents a more spatially dispersed distribution of fixations across the AOIs. GTE represents the overall uncertainty associated with the temporal sequence of fixations, given the current fixation location (i.e., AOI), (Equation 2). Specifically, a higher GTE value indicates that gaze scan paths are more complex and frequently cross various AOIs in varying order throughout task completion.

**Figure 5.**
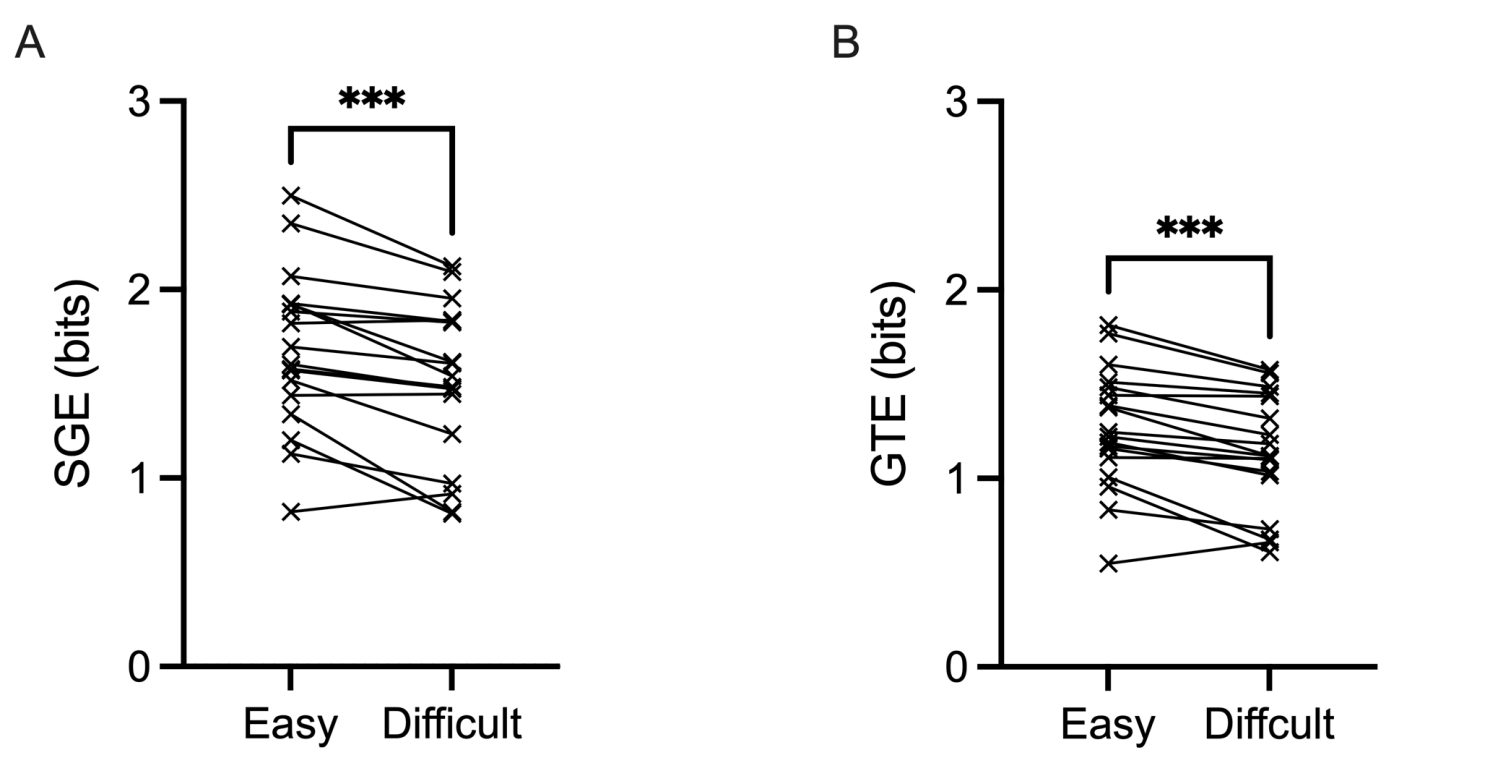
Individual data points for stationary gaze entropy (SGE) (bits) (A) and gaze transition entropy (GTE) (B) are demonstrated for easy and difficult conditions. Error bars represent SEM. *p ≤0.05, **p ≤0.01, ***p ≤0.001, ****p ≤0.0001.

SGE revealed a main effect of task difficulty, *F*(1,17)=20.898, *p*<0.001, 𝜂_p_^2^=0.551. Specifically, the spatial distribution of fixations was significantly more dispersed during easy trials (mean=1.7 bits, SD=0.4 bits) compared to difficult trials (mean=1.5 bits, SD=0.4 bits) (Figure 5A). Additionally, GTE produced a main effect of task difficulty, *F*(1,17)=23.986, *p*<0.001, 𝜂_p_^2^=0.585. Figure 5B shows how the overall gaze sequence was more complex due to an increase in AOIs being fixated in a more random sequence during easy trials (mean=1.3 bits, SD=0.3 bits) and became more predictable during difficult trials (mean=1.1 bits, SD=0.3 bits).

**Figure 6.**
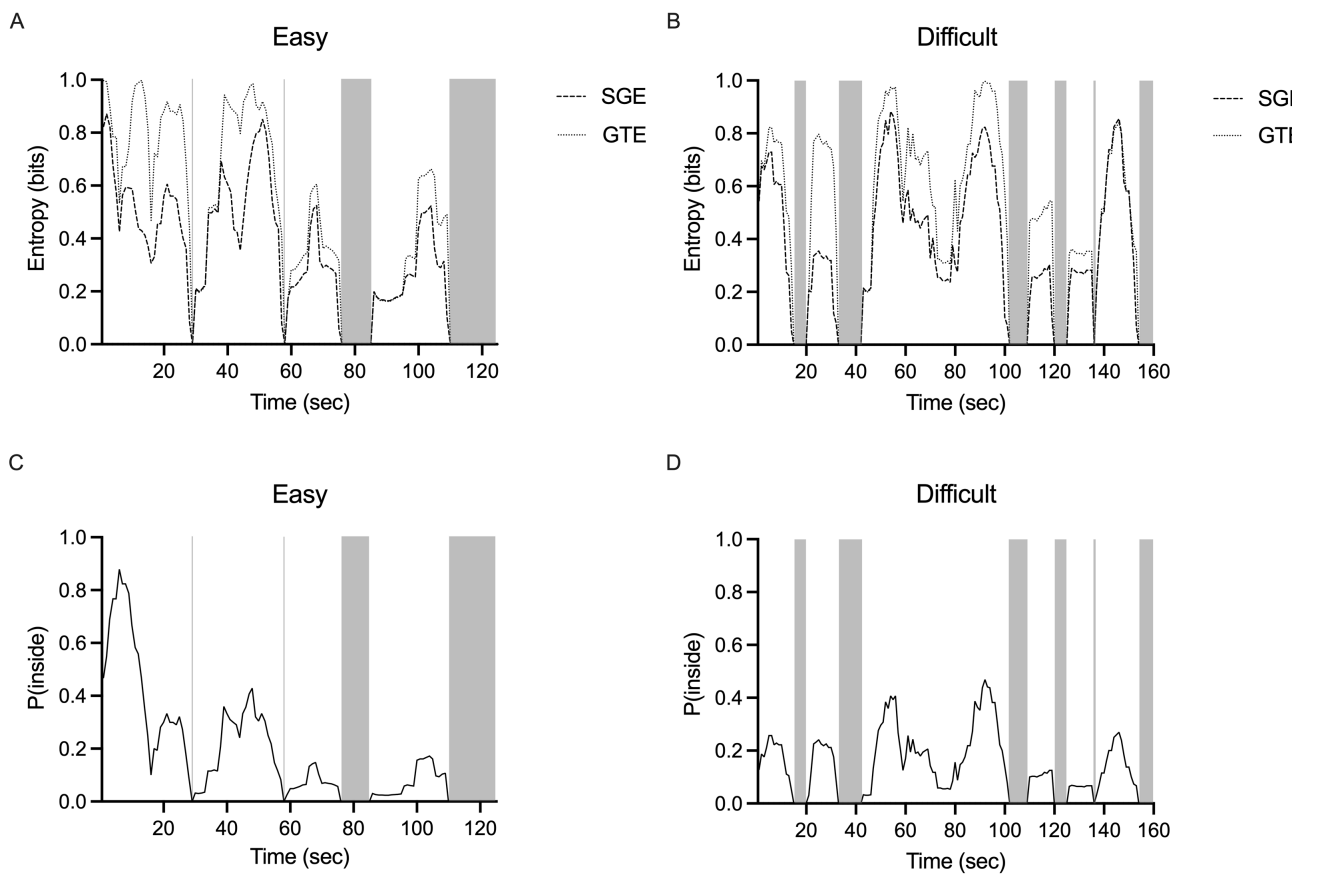
Representative time traces that reflect the stationary gaze entropy (SGE) and gaze transition entropy (GTE) of a single easy (A) and difficult (B) trial. Exemplar time traces showing the probability of fixating inside the cockpit for the easy (C) and difficult (D) conditions. These reflect the same easy (A and C) and difficult (B and D) trials. Gray boxes represent the sections of the trial where entropy and P(inside) equals zero. This indicates that gaze is directed outside the cockpit for at least 10 seconds (i.e., a single bout). The easy condition illustrates four bouts with a total bout time of 68 sec (A). The difficult condition illustrates six bouts with a total bout time of 96 sec (B).

Video recordings of participants’ eye movements demonstrated that some participants adopted a gaze pattern that reflects a continuous allocation of attention outside of the cockpit, toward the runway. In contrast, other participants showed a gaze pattern that continually cycled through the various AOIs inside and outside the cockpit. Similar observations have been reported in other work (5; 72); however, they were not objectively quantified. Therefore, additional analyses were completed to further examine how gaze entropy changed over the course of a trial. A 30 second average sliding window was initially chosen arbitrarily (60); however, we found it did not have sufficient resolution to characterize the dynamic cyclical nature of the gaze patterns that were apparent in video recordings of participants’ gaze. As such, we used a 10 second average sliding window to produce a time trace that reflected how SGE and GTE evolved over time. To further characterize the observed pattern of switching gaze between the internal cockpit environment and external scenery, all cockpit AOIs (i.e., 1-7) were collapsed into a single ‘inside’ AOI and all external AOIs (8-10) were collapsed into as a single ‘outside’ AOI. The resulting entropy traces provide a temporal window depicting gaze dispersion and sequence predictability changes as participants completed the landing task (Figure 6AB).

Examining the SGE and GTE time traces revealed moments where both entropy measures fell to zero, which indicates that gaze was directed either outside or inside the cockpit for at least 10 seconds (Figure 6AB). To determine if the reduction in gaze entropy to zero reflected gaze allocation inside or outside the cockpit, we calculated the probability of a fixation being inside the cockpit over the length of the trial, P(inside) (Figure 6CD). Specifically, each fixation was assigned a binary number based on whether the fixation was inside or outside the cockpit. P(inside) was then computed as the number of fixations inside the cockpit divided by the total number of fixations in the 10 second window. When P(inside) was equal to 1, the participant was continuously fixating inside the cockpit for at least 10 seconds. When P(inside) was equal to 0, the participant was continuously fixating on the outside scenery for at least 10 seconds (Figure 6CD). This analysis provides an objective approach to monitor the temporal dynamics of gaze behaviour, which may reflect attention and how it is deployed inside and outside the cockpit. More specifically, the ‘bout’ analysis served to pinpoint the times when pilots stopped cycling through both internal and external environments. Given the time series of fixation probabilities showed distinct periods of fixations outside the cockpit, a ‘bout’ was defined as a period of time in which fixations remained entirely outside of the cockpit for at least 10 seconds. These bouts were detected as connected components (subsequent values) of zeros in the probability time series. Number of bouts was defined as the number of instances a bout was detected within a trial. Bout duration (sec) was defined as the average duration of all bouts that occurred in a trial. Total bout time (sec) was defined as the sum of all the individual bout durations within a trial.

**Figure 7.**
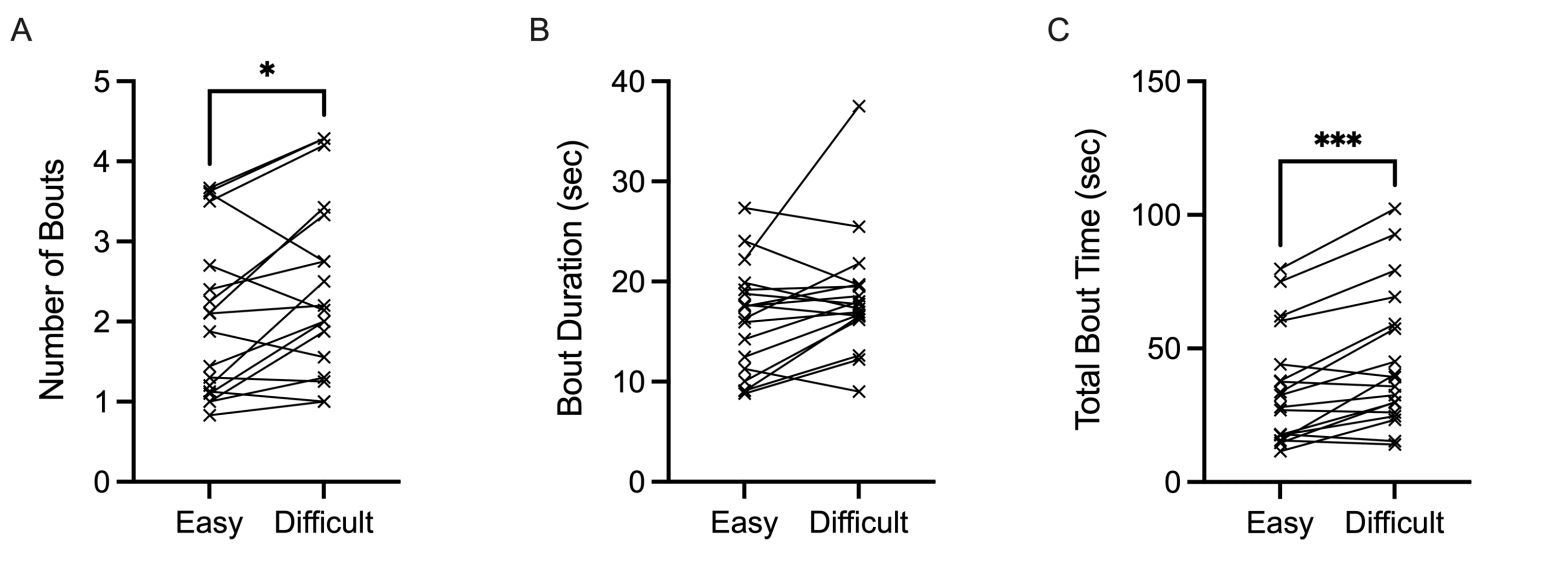
Individual data points for number of bouts (A), bout duration (sec) (B), and total bout time (sec) (C) are demonstrated for easy and difficult conditions. *p ≤0.05, **p ≤0.01, ***p ≤0.001, ****p ≤0.0001.

Number of bouts yielded a main effect of task condition, *F*(1,17)=7.224, *p*=0.016, 𝜂_p_^2^=0.298. Specifically, easy trials (mean=2.0, SD=1) were associated with fewer bouts than difficult trials (mean=2.4, SD=1) (Figure 7A). Average bout duration (sec) was not significantly impacted by task difficulty, *F*(1,17)=4.233, *p*=0.055 (Figure 7B). Last, total bout time (sec) was also significantly influenced by task difficulty, *F*(1,17)=20.317, *p*<0.001, 𝜂_p_^2^=0.544 (Figure 7C). Difficult trials (mean=45 sec, SD=26 sec) were associated with more continuous fixation outside of the cockpit compared to easy trials (mean=35 sec, SD=21 sec).

## Discussion

This study characterized the effects of task difficulty on gaze behaviour and flight performance in low-time pilots as they completed simulated landing scenarios. Participants were asked to perform the landing task in high visibility, visual flight rules (VFR) conditions that differed based on the presence (difficult) or absence (easy) of strong winds. Several notable contributions emerged from this study. First, in support of our hypothesis, a comprehensive assessment of gaze behaviour during landing revealed that dwell time and entropy-based measures were modulated by changes in task difficulty. Specifically, increasing task difficulty resulted in longer fixation of the runway, and a reduction in the dispersion (SGE) and complexity (GTE) of gaze sequences. Second, further exploration of the data led to a development of a novel approach to objectively identify and track instances when pilots selectively allocate their attention outside the cockpit.

### The effect of task difficulty on performance

The analysis of landing performance provided unambiguous evidence about the successful manipulation of task complexity. Specifically, when dealing with strong wind conditions, pilot performance decreased. A finding that was made evident through a reduction in landing success rate and overall performance score, alongside an increase in completion time and landing accuracy error. Although a formal examination of subjective perception of task difficulty was not conducted in the current study, participants reported that they noticed an increase in task difficulty during the trials that involved turbulent weather conditions. Overall, these results are in line with earlier studies using similar experimental procedures (21).

### The effect of task difficulty on pilot’s gaze behaviour

The examination of gaze behaviour via traditional gaze metrics provides a proxy for the allocation of attention toward task relevant information and how it is used to facilitate task performance (24; 29; 38; 56; 73). The current study demonstrated that task difficulty was associated with a significant increase in the time spent looking outside toward the runway and a corresponding reduction in the time spent fixating on alternative AOI’s that included the airspeed indicator, the power indicator, and the horizon. These findings suggest that attention was focused on the runway during strong wind conditions at the expense of other AOIs both within and outside of the cockpit. In line with these findings, average saccade amplitude and variability also decreased as a function of task difficulty. A finding that indicates a reduction in the spread of sequential fixation locations, and hence a focusing of visual attention to a limited set of closely spaced regions within the task environment. Notably, the addition of strong wind conditions introduced a significant crosswind component which required continuous monitoring of heading direction during final approach and landing. The use of the crab or sideslip methods to compensate for the crosswind may have led participants to look outside more, not only to check on wind conditions but to monitor the plane’s trajectory and ensure that the glideslope and heading were maintained and adjusted accordingly (26). This is further supported by other work demonstrating that the allocation of attention outside the cockpit in VFR conditions helps provide the pilot with relevant visual information that facilitates successful landing (9; 24; 29; 38; 50; 56). Though fixation rates did not increase as a function of task difficulty across any AOIs, they did decrease for the airspeed indicator and the horizon. Further demonstrating that the scanning of information in these AOI’s was reduced at the cost of spending more time fixating on or around the runway, which contributed to the reduction in dwell time percentages in these respective AOIs. These findings echo previous work by Van de Merwe et al. (64), which found that fixation rates on various instruments were related to their problem-relevance; with the runway being of prime importance (14; 46). Interestingly, a study by Babu et al. (7) demonstrated that fighter pilots increased their fixation frequency when task difficulty increased during a longitudinal target tracking task, while Tole et al. (63) found no change in the fixation rate of military pilots associated with turbulence in a landing task. The contradictory findings may lie in the task employed (7) and the role that experience plays in the gaze behaviour supporting performance (7; 63). For instance, experienced pilots tend to fixate relevant cockpit gauges for briefer periods of time more frequently compared to novice pilots (28). Additionally, our fixation rate does correct for the increased time spent completing the landing task during the turbulent conditions, whereas previous studies simply reported the fixation frequency count (31; 46; 61). Taken together, our traditional eye movement analysis measures suggest that the allocation of attention in low-time pilots became more biased toward the runway in order to monitor and extract the necessary information needed to land during challenging turbulent flight scenarios.

The analysis of gaze entropy is a surrogate for exploring the dynamic nature of gaze behaviour. Specifically, the dispersion (SGE) and sequence (GTE) of a scanning pattern have been shown to be effective indicators of attention deployment (i.e., focal versus exploratory modes of attention) as well as the complexity of the scanning pattern structure (59). Indeed, previous work has shown that the competition of bottom-up (salience driven) and top-down (executive control driven) attentional processes influence the spatial prioritization of where we look (11; 25; 1Fecteau and Munoz, 2006; 59). For instance, bottom-up processes would likely drive attention to salient objects in the environment which may correspond to more random and dispersed fixation patterns (59). Whereas top-down input would direct attention based on task knowledge, expectations, and current goals, and thus reflect task engagement via a reduction in the dispersion of fixations and more structured scanning patterns (59). The current findings revealed that low-time pilot’s gaze entropy became less dispersed and less complex during strong wind conditions. That is, pilots followed a more predictable and deterministic visual scanning pattern during the more complex landing scenarios. This is analogous to previous studies that also reported an increase in the scanning and allocation of attention to a limited set of task critical AOIs during more complex landing scenarios (31; 70). Notably, these findings are similar to those seen using the saccade amplitudes metrics (43). However, in addition to being able to capture the spread of fixations across task-relevant areas of interest that are object-defined (i.e., the runway, side window, specific cockpit gauges) the secondary entropy measure, GTE, helps to characterize the complexity of the scanning sequence itself within the task environment. This provides critical information regarding visual scanning patterns that can differ significantly despite occupying the same spatial regions. In line with previous work, our findings support the notion that pilots use exploratory and saliency-driven gaze patterns when the aircraft is in an error-free state (i.e., perfect flying conditions) and change their gaze behaviour when they are experiencing periods of flight that involve more challenging task demands; a shift that reflects the top-down attentional control imposed on visual scanning to help focus attention to the appropriate object at the appropriate time (4; 10; 13; 25; 32; 59; 63). It is also important to note that additional entropy analysis was conducted to ensure that AOI size disparities across the 10 defined regions did not bias the entropy results. We calculated entropy using the two AOIs (outside vs. inside), which resulted in a 50-50 screen split of equally sized AOIs. Indeed, the main effects reported in the current study were maintained (all *ps*<0.02) and support that our entropy findings were not biased by AOI size.

The current study makes an important contribution by quantifying the spatiotemporal gaze dynamics using a novel approach. Previous work reported an observation that novice pilots tend to restrict their gaze toward the external view of the cockpit and to focus predominantly on the runway, which has been referred to as ‘gaze tunneling’ (72; 73). Unfortunately, previous studies did not quantify this behaviour objectively. We adopted a sliding window approach to quantify the moment-to-moment changes in gaze entropy and the probability of fixating inside and outside the cockpit. This approach enabled the quantification and analysis of runway tunneling ‘bouts’ – that is, the continuous fixation of attention outside of the cockpit toward the runway, which were then examined with respect to their relationship with pilot performance and task difficulty. Our results demonstrated that an increase in task difficulty was associated with a greater number of bouts as well as an increase in total bout time. Therefore, strong wind conditions resulted in low-time pilots becoming more stringent in attending to information outside of the cockpit- for intervals greater than 10 seconds- instead of inside toward the cockpit instrument panel; a finding taken to evince less cycling of attention between the external and internal cockpit environments. Notably, this may provide an alternative explanation for why entropy values decrease during more difficult trials. That is, instead of visual scanning becoming more structured and deterministic, it may be that SGE and GTE values are reduced due to an increase in gaze tunneling; an indicator of poor monitoring. In this scenario, it is expected that these gaze tunneling events will impair task performance. Though there were not enough trials to conduct a statistical analysis of these characteristic runway tunnelling bouts on failed landing trials, a preliminary analysis of these rare landing failures demonstrated longer bout durations and total bout time during the early part of the flight in these trials. Additionally, we found that a group of less experienced low-time pilots (n=11) had more bouts of fixated attention outside the cockpit and longer total bout time compared to low-time pilots who had at least obtained their private pilot’s licence (PPL) (n=7). Notably, the analysis of gaze behaviour profiles has not been examined in these smaller experience increments, and are being further investigated in a subsequent study. These initial findings suggest that the exclusive fixation of attention toward the runway for extended periods of time may impact pilots’ situational awareness and mental models (5; 26; 72). Though, neither of these outcomes would necessarily lead to poor landing performance when the plane is in an error-free state, this could pose a significant problem to landing performance and safety should an emergency/error arise. Further investigation of gaze behaviour is required as it could potentially lead to the development of an objective method allowing an ‘online’ detection of pilot inattention, poor pilot monitoring, or a general marker of divergence from optimal scanning.

### Limitations

This study provides several notable contributions regarding how task difficulty may influence gaze behaviour. However, the current results are constrained by at least two limitations that should be addressed in future research. First, the methodological challenges imposed by the Microsoft Flight Simulator gaming environment limited real-time synchronization and made it difficult to understand the relationship between game generated landing performance scores and their ecological significance. Additional research using other simulator environments is needed to address this issue. For instance, it is crucial to address the basic relationship and temporal contingency between specific eye movements and actions (i.e., vision-in-action paradigms) to determine whether specific types of gaze characteristics are related to superior performance of specific actions. Alternatively, this could also allow for the exploration of specific types of gaze characteristics that lead to poor outcomes (i.e., accidents, unstable approaches, loss of control). Second, we compared low-time pilot performance during an error-free state landing scenario in VFR conditions to that during strong wind conditions. It may be important to consider exploring the gaze profile differences between different classes of low-time pilots (i.e., those who have at least obtained their PPL and those who have not). Further research should take these smaller intervals of expertise into account starting from the ab initio stage (i.e., little to no flying experience) up to expert (i.e., every new licensing level- PPL, Commercial Pilot’s License, etc.) with much larger sample sizes (i.e., >15 pilots per group). This will allow for a finer characterization and examination of gaze behavior evolution and skill progression throughout the course of pilot training. This remains a crucial gap in the literature because little is known about the role that gaze behaviour plays in early skill learning and progression in complex environments.

### Conclusion

This work highlighted the performance and gaze differences in low-time pilots when completing a simulating landing scenario in VFR conditions with and without turbulence. During turbulent weather conditions, pilots shifted their gaze behaviour to become less complex and more focused on the runway, thus making the scanning and processing of information more targeted towards fewer areas of interest. Overall, the gaze-related metrics used in the present study provide valuable information for assessing pilot gaze behaviour in the cockpit and can contribute to better characterization of visual scanning. This remains an important area of research because understanding how gaze contributes to optimal pilot performance might be an important benchmark for monitoring and ensuring flight safety as well as evaluating pilot competency during training.

### Ethics and Conflict of Interest

The author(s) declare(s) that the contents of the article are in agreement with the ethics described in http://biblio.unibe.ch/portale/elibrary/BOP/jemr/ethics.html and that there is no conflict of interest regarding the publication of this paper.

### Acknowledgements

This research was supported in part by grant 00753 from the New Frontiers in Research Fund.
